# Temporal regulation of protein *O*‐GlcNAc levels during pressure‐overload cardiac hypertrophy

**DOI:** 10.14814/phy2.14965

**Published:** 2021-08-02

**Authors:** Wei Zhong Zhu, Dolena Ledee, Aaron K. Olson

**Affiliations:** ^1^ Seattle Children’s Research Institute Seattle WA USA; ^2^ Division of Cardiology Department of Pediatrics University of Washington Seattle WA USA

**Keywords:** Cardiac hypertrophy, GFAT, glucose metabolism, hexosamine biosynthesis pathway, *O*‐GlcNAc, pressure‐overload hypertrophy, thiamet‐g, transverse aortic constriction

## Abstract

Protein posttranslational modifications (PTMs) by *O*‐linked β‐*N*‐acetylglucosamine (*O*‐GlcNAc) rise during pressure‐overload hypertrophy (POH) to affect hypertrophic growth. The hexosamine biosynthesis pathway (HBP) branches from glycolysis to make the moiety for O‐GlcNAcylation. It is speculated that greater glucose utilization during POH augments HBP flux to increase *O*‐GlcNAc levels; however, recent results suggest glucose availability does not primarily regulate cardiac *O*‐GlcNAc levels. We hypothesize that induction of key enzymes augment protein *O*‐GlcNAc levels primarily during active myocardial hypertrophic growth and remodeling with early pressure overload. We further speculate that downregulation of protein O‐GlcNAcylation inhibits ongoing hypertrophic growth during prolonged pressure overload with established hypertrophy. We used transverse aortic constriction (TAC) to create POH in C57/Bl6 mice. Experimental groups were sham, 1‐week TAC (1wTAC) for early hypertrophy, or 6‐week TAC (6wTAC) for established hypertrophy. We used western blots to determine *O*‐GlcNAc regulation. To assess the effect of increased protein O‐GlcNAcylation with established hypertrophy, mice received thiamet‐g (TG) starting 4 weeks after TAC. Protein *O*‐GlcNAc levels were significantly elevated in 1wTAC versus Sham with a fall in 6wTAC. OGA, which removes *O*‐GlcNAc from proteins, fell in 1wTAC versus sham. GFAT is the rate‐limiting HBP enzyme and the isoform GFAT1 substantially rose in 1wTAC. With established hypertrophy, TG increased protein *O*‐GlcNAc levels but did not affect cardiac mass. In summary, protein *O*‐GlcNAc levels vary during POH with elevations occurring during active hypertrophic growth early after TAC. *O*‐GlcNAc levels appear to be regulated by changes in key enzyme levels. Increasing *O*‐GlcNAc levels during established hypertrophy did not restart hypertrophic growth.

## Graphical abstract

Protein *O*‐GlcNAc levels temporally vary during pressure‐overload hypertrophy (POH) with the highest levels during active hypertrophic growth early after transverse aortic constriction. While it is commonly hypothesized that *O*‐GlcNAc levels reflect increased glucose utilization during POH, our study confirms the importance of non‐nutrient regulation through enzymes like GFAT1 and OGA.

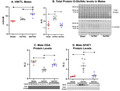

## 1 INTRODUCTION

Posttranslational modification (PTM) of serine/threonine protein residues by *O*‐linked β‐*N*‐acetylglucosamine (*O*‐GlcNAc) is a dynamic and reversible process affecting protein functions (Hanover et al., 2010; Torres & Hart, 1984). Protein O‐GlcNAcylation affects many cellular functions including cell cycle regulation, transcriptional and translational events, metabolism, mitochondria function, protein synthesis and quality control, autophagy, epigenetic signaling, and calcium handling (Marsh et al., 2014; Wright et al., 2017). Total protein *O*‐GlcNAc levels rise during cardiac pathologies including hypertrophy and heart failure in both humans and animal models (Dassanayaka et al., 2017; Gélinas et al., 2018; Lunde et al., 2012; Tran et al., 2020; Watson et al., 2010; Zhu et al., 2019). However, a lack of knowledge regarding the dynamic regulation of protein *O*‐GlcNAc levels impedes our understanding of its role during cardiac hypertrophy.

**FIGURE 1:**
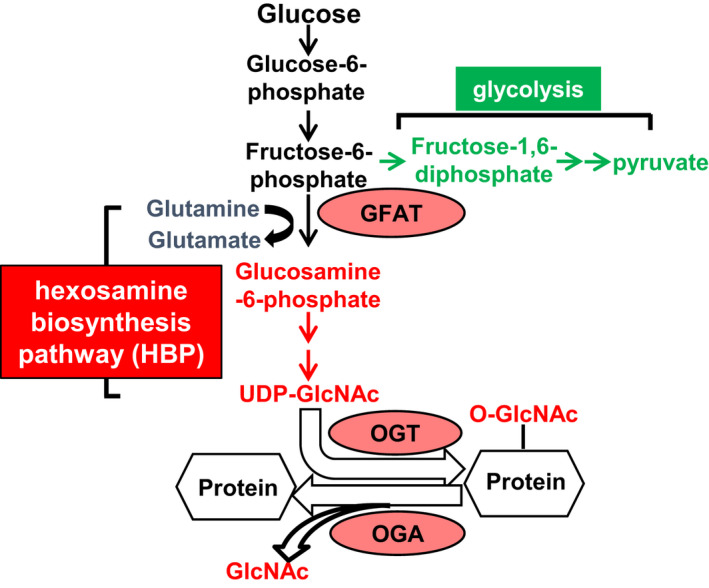
The hexosamine biosynthesis pathway (HBP) and glycolysis. This figure shows important metabolic intermediates for these pathways as well as the main enzymes for the HBP and protein O‐GlcNAcylation. Intermediates shared between glycolysis and HBP are shown in black, HBP‐only intermediates are shown in red, and glycolysis‐only intermediates are shown in green. Glucose metabolized to fructose‐6‐phosphate (F6P) can enter the HBP or undergo further glycolysis to generate pyruvate. GFAT (enzymes shown in ovals) is the rate‐limiting enzyme in the HBP and uses glutamine to catalyze the conversion of F6P to glucosamine‐6‐phosphate. After a series of reactions, uridine diphosphate‐β‐GlcNAc (UDP‐GlcNAc) is the end product of the HBP and serves as the donor for the enzyme *O*‐GlcNAc transferase (OGT) to perform *O*‐GlcNAc protein post‐translational modifications. The enzyme *O*‐GlcNAcase (OGA) removes the GlcNAc moiety from proteins. Pyruvate is the end product of glycolysis

Differing from most PTMs, protein O‐GlcNAcylation is regulated by a single enzyme for attachment (*O*‐GlcNAc transferase, OGT), and a single enzyme for removal (*O*‐GlcNAcase, OGA) (Figure 1). Thus, *O*‐GlcNAc levels are thought to change in a more coordinated manner than many other PTMs. Protein *O*‐GlcNAc levels are also determined by flux through a branch pathway from glycolysis called the hexosamine biosynthesis pathway (HBP). Glucose metabolized to fructose‐6‐phosphate (F6P) can enter the HBP or continue through glycolysis to generate pyruvate (Figure 1). L‐glutamine:fructose‐6‐phosphate amidotransferase (GFAT) is the rate‐limiting HBP enzyme and uses glutamine to catalyze the conversion of F6P to glucosamine‐6‐phosphate. UDP‐GlcNAc is the final product of the HBP and serves as the donor for OGT to perform *O*‐GlcNAc PTMs. OGT is sensitive to UDP‐GlcNAc concentration, making overall protein *O*‐GlcNAc levels responsive to changes in HBP flux (Kreppel & Hart, 1999). Given the link between glycolysis and the HBP, it is widely assumed that protein *O*‐GlcNAc levels passively increase secondary to greater glucose availability from augmented glycolytic flux during pressure‐overload cardiac hypertrophy (POH) (Dassanayaka & Jones, 2014; Mailleux et al., 2016; Marsh et al., 2014; Wright et al., 2017).

Recent results, however, raise fundamental questions regarding regulation of the HBP and protein *O*‐GlcNAc levels during POH. First, we found that HBP flux remains constant even through a 20‐fold change in glycolytic rates in non‐hypertrophied hearts. Thus, our result implies that glycolytic rates do not primarily regulate HBP flux and *O*‐GlcNAc levels (Olson et al., 2020). Second, Tran et al., (2020) found that GFAT1 isoform induction augments protein *O*‐GlcNAc levels after transverse aortic constriction (TAC). Cardiac‐specific GFAT1 knockout prior to TAC reduced *O*‐GlcNAc levels. Additionally, GFAT1 knockout reduced hypertrophic growth in their studies. Interestingly, our prior work suggested that protein *O*‐GlcNAc levels vary during pressure‐overload hypertrophy with the highest levels during active hypertrophic growth (Zhu et al., 2019). Thus, these findings suggest that the HBP and protein O‐GlcNAcylation are temporally regulated to promote hypertrophic growth and remodeling early after TAC.

We hypothesize that key regulatory enzymes such as GFAT, OGT, and OGA are induced to increase protein O‐GlcNAcylation primarily during active myocardial hypertrophic growth and remodeling early after TAC. We further speculate that downregulation of protein O‐GlcNAcylation inhibits ongoing hypertrophic growth during prolonged pressure‐overload. To test our hypothesis, we evaluated hearts during rapid hypertrophic growth early after TAC (1 week after TAC), as well as late after TAC (6 weeks after TAC) when hypertrophic growth stabilizes (established hypertrophy). We secondarily tested whether augmenting protein *O*‐GlcNAc levels during established hypertrophy could restart hypertrophic growth.

## 2 MATERIALS AND METHODS

### 2.1 Ethics statement

This investigation conforms to the Guide for the Care and Use of Laboratory Animals published by the National Institute of Health (NIH Pub. No. 85‐23, revised 1996) and were reviewed and approved by the Office of Animal Care at Seattle Children's Research Institute. All materials used in this study are available commercially from the indicated vendors.

### 2.2 Mice

The primary studies were performed in male and female C57BL/6J mice originating from the Jackson Laboratory (Bar Harbor, ME) between the ages of 3 and 5 months. Cardiac hypertrophy differs between males and females; therefore, the genders were evaluated separately. All mice were allowed free access to water and Teklad #7964 chow (Envigo). Teklad ¼ inch corncob bedding was utilized in the cages (Envigo).

### 2.3 Experimental setup

We hypothesize that key regulatory enzymes are induced to increase protein O‐GlcNAcylation primarily during active myocardial hypertrophic growth early after imposition of aortic constriction. Second, we speculate that downregulation of protein O‐GlcNAcylation inhibits ongoing hypertrophic growth during prolonged pressure‐overload with established hypertrophy. TAC surgery causes rapid (active) hypertrophic growth early after imposition of aortic constriction, whereas cardiac mass remains relatively stable with prolonged TAC. Thus, to test our hypothesis, we performed TAC to create pressure‐overload hypertrophy and assessed hearts either during active hypertrophic growth 1 week after TAC (referred to as 1wTAC) or during prolonged pressure overload when hypertrophic growth has stabilized (established hypertrophy) 6 weeks after TAC (referred to as 6wTAC). In preliminary studies (data not shown), we found similar protein levels of *O*‐GlcNAc, OGT, OGA, and GFAT1/2 for sham‐operated hearts at 1 week versus 6 weeks. Thus, we combined both sham durations into a single sham group for comparisons, which also allowed for a reduction in the total number of mice utilized for these experiments. The groups for these experiments are sham, 1wTAC representing the period of active hypertrophic growth and 6wTAC representing the period when hypertrophic growth has stabilized (also referred to as established hypertrophy).

To determine whether increasing protein *O*‐GlcNAc levels could restart hypertrophic growth during established hypertrophy, we gave daily thiamet‐g (TG) or vehicle (V) injections to C57BL/6J mice starting 4 weeks after TAC and continuing until 6 weeks post‐TAC. The groups for these experiments are TG‐TAC and V‐TAC.

Female gender modifies both cardiac hypertrophy and metabolism (Fliegner et al., 2010; Taegtmeyer et al., 2016; Weinberg et al., 1999), so we separately evaluated female and male mice in our experiments. To our knowledge, no prior studies including our own assessed protein O‐GlcNAcylation during POH in female versus male mice (Dassanayaka et al., 2017; Tran et al., 2020; Zhu et al., 2019).

### 2.4 Transverse aortic constriction surgery (TAC)

We titrated the degree of aortic constriction with our TAC surgery to create pathological POH with relatively stable cardiac systolic function between 1wTAC and 6wTAC. The surgery was performed as previously described in our lab with a few changes (Ledee et al., 2015; Zhu et al., 2019). Briefly, mice were initially anesthetized with 3% isoflurane in 100% O_2_ at a flow of 1 LPM and then maintained with 1.5% isoflurane for the duration of the surgery. A midline sternotomy was performed to expose the aorta. The aorta was constricted with 7‐0 silk suture tied against a 22‐gauge blunt needle for males and a 23‐gauge blunt needed for females (C57Bl6/J). Our preliminary studies showed a similar degree of aortic constriction (measured by echocardiogram) between the genders with this approach. Sham mice underwent similar surgery but did not have the suture tightened around the aorta. For pain control, all mice received intraperitoneal buprenorphine (0.05–0.1 mg/kg IP) starting preoperatively and continuing approximately every 12 h for 2 days.

### 2.5 Thiamet‐G (TG)

We used the OGA inhibitor TG (Cayman Chemical Company) at 20 mg/kg/day once daily for 2 weeks intraperitoneal to increase protein *O*‐GlcNAc levels. TG was prepared in dimethyl sulfoxide (approximately 20 μl) and control mice received an equivalent volume of the vehicle only.

### 2.6 Echocardiogram

Echocardiograms were performed as previously described in our lab (Ledee et al., 2015; Olson et al., 2013; Standage et al., 2017; Zhu et al., 2019). Briefly, mice were initially sedated with 3% isoflurane in O_2_ at a flow of 1 LPM and placed in a supine position at which time the isoflurane is reduced to 1.5% administered via a small nose cone. ECG leads were placed for simultaneous ECG monitoring during image acquisition. Mice were maintained a temperature of 37.5℃ throughout the echocardiogram. Echocardiographic images were performed with a Vevo 2100 machine using a MS250 or MS550 transducers (VisualSonics, Inc). M‐Mode measurements at the midpapillary level of the left ventricle (LV) were performed at end‐diastole (EDD) and end‐systole (ESD) to determine LV function via the fractional shortening [(LVEDD‐LVESD)/LVEDD × 100] in a parasternal short axis mode for at least three heart beats. The pulsed Doppler velocity was measured across the aortic constriction site. This velocity serves as a noninvasive estimate of the peak instantaneous pressure gradient across the constriction site as calculated by the simplified Bernoulli equation (Δ pressure gradient = 4 × velocity^2^). The echocardiogram reader was blinded to treatment. We previously found that isoflurane anesthesia during echocardiograms increases myocardial total protein *O*‐GlcNAc levels for several hours after the procedure (unpublished data). Therefore, the final echocardiograms were always performed on the day prior to sacrifice.

### 2.7 Western Blots

Immunoblots were performed as previously described on freshly isolated hearts (Ledee et al., 2015; Olson et al., 2013; Standage et al., 2017; Zhu et al., 2019). Briefly, protein was isolated with a lysis buffer containing 10 mM Tris (pH = 7.6), 150 mM NaCl, 0.5% deoxycholate, 0.1% SDS, 1% NP‐40 containing phosphatase inhibitor (ThermoFisher Scientific) plus the OGA inhibitors PUGNAc 20 µM (Toronto Research Chemical) and Thiamet‐G 1µM (Adooq Bioscience). Fifty micrograms of total protein extract from mouse heart tissue was separated electrophoretically and transferred to PVDF membrane. The membranes were probed with the following antibodies: OGT (#5368, Cell Signaling Technologies), OGA (NBP1‐81244, Novus Biologicals), GFAT1 (ab125069, Abcam), GFAT2 (sc134710, Santa Cruz Biotechnology), and activating transcription factor 4 (ab184909, ATF4, Abcam). Membranes were stripped by washing for 30 min with 100 mM dithiothreitol, 2% (wt/vol) SDS, 62.5 mM Tris·HCl, pH 6.7, at 70℃, followed by three 10‐min washes with TBS for additional antibody analysis. Immunoblots were normalized to total protein staining in the molecular weight region around the band of interest by Thermo Scientific Pierce Reversible Protein Stain Kit for PVDF Membranes (Thermo Scientific), which are shown in the figures. Immunoblots and protein staining were measured by densitometry analysis using ImageJ software (National Institute of Health).

The *O*‐GlcNAc western blots require additional details from the general immunoblot methods described above. Our unpublished data shows that isoflurane increases myocardial O‐GlcNAcylation for hours after an exposure; therefore, all mice underwent sedated echocardiograms on the day prior to tissue collection. The protein lysis buffer included the OGA inhibitors PUGNAc and TG (concentrations noted above). Protein *O*‐GlcNAc levels were primarily determined with the RL‐2 antibody from Abcam (ab2739). We confirmed total protein *O*‐GlcNAc in sham, 1wTAC, and 6wTAC with CTD110.6 antibody (9875s, Cell Signaling Technologies), with is another commonly used antibody for determining *O*‐GlcNAc levels. Both of these antibodies are raised in mice and the secondary anti‐mouse antibody can react with IgG from the tissue resulting in a nonspecific band at either 55 kD (from IgG heavy chain) or 25 kD (from IgG light chain). To allow for unobstructed detection of *O*‐GlcNAc bands in the 55‐kD region, we only used a light chain‐specific secondary antibody (Peroxidase AffiniPure goat anti‐mouse IgG, light chain specific; Jackson ImmunoResearch Laboratories, Inc.) for our *O*‐GlcNAc immunoblots and excluded the 25‐kD band during quantification. Total (global) *O*‐GlcNAc levels were measured by densitometry of the entire western blot lane. *O*‐GlcNAc levels were normalized to the protein staining by Thermo Scientific Pierce Reversible Protein Stain Kit for PVDF Membranes (described above) of the entire lane or same molecular weight region as appropriate.

### 2.8 Total RNA extraction, reverse‐transcription and semi‐quantitative RT‐PCR for X‐box‐binding protein 1 splice variant (XBP1s), natriuretic peptide A (Nppa), and natriuretic peptide B (Nppb)

Total mRNA was isolated from the frozen heart tissue using RNeasy Fibrous Tissue Mini Kit (Qiagen). DNase I was used to remove genomic DNA. Total RNA concentration and purity were measured on a NanoDrop spectrophotometer (ThermoFischer Scientific). Next, 1 μg of RNA was reverse transcribed into cDNA using the High Capacity cDNA Reverse Transcription kit (Applied Biosystem, ThermoFischer Scientific). 20 ng of the resulting cDNA was amplified by standard PCR with TaqDNA polymerase (Invitrogen, ThermoFischer Scientific). The XBP1s primers were used as described previously (Wang et al., 2014). Mouse *Nppa* sense primer sequence: 5’AATAAACTTCAGCACCAAGGAC3’ and antisense primer sequence: 5’GTCCTTGGTGCTGAAGTTTATT3’ (Pedernera et al., 2017). Mouse *Nppb* sense primer sequence: 5’GTCCAGCAGAGACCTCAAAA3’ and antisense primer sequence: 5’AGGCAGAGTCAGAAACTGGA3’ (Sergeeva et al., 2014). Beta‐actin (forward 5’AGTTCTACAAATGTGGCTGAGGA3’, reverse 5’GGTAAGGTGTGCACTTTTATTGG3’) was used as a loading control. Thermocycling condition were: Denaturation at 94˚C for 30 s, annealing at 58˚C for 30 s, extension at 72˚C for 30 s, for 25 cycles. The products were run in 1% agarose gel with SYBR Safe DNA Gel Stain (Invitrogen, ThermoFischer Scientific), and images were captured with ChemiDoc‐It^2^ (Analytik Jenna US LLC). For analysis, we used the fold change in Nppa or Nppb over the average of the sham group (for sham, 1wTAC, and 6wTAC experiments) or V‐TAC (for the TG experiments).

### 2.9 Data visualization and statistical analysis

We preformed the statistical analysis and data visualization using GraphPad Prism version 8.3.3 (GraphPad Software). All reported values are mean ± standard error of the mean (SEM). An unpaired *t*‐test was used for comparisons of two groups. For comparisons of multiple groups, we utilized a one‐factor ANOVA with a Tukey's correction for multiple procedures. Criterion for significance was *p *< 0.05.

## 3 RESULTS

### 3.1 Echocardiographic and morphometric results during POH

**FIGURE 2:**
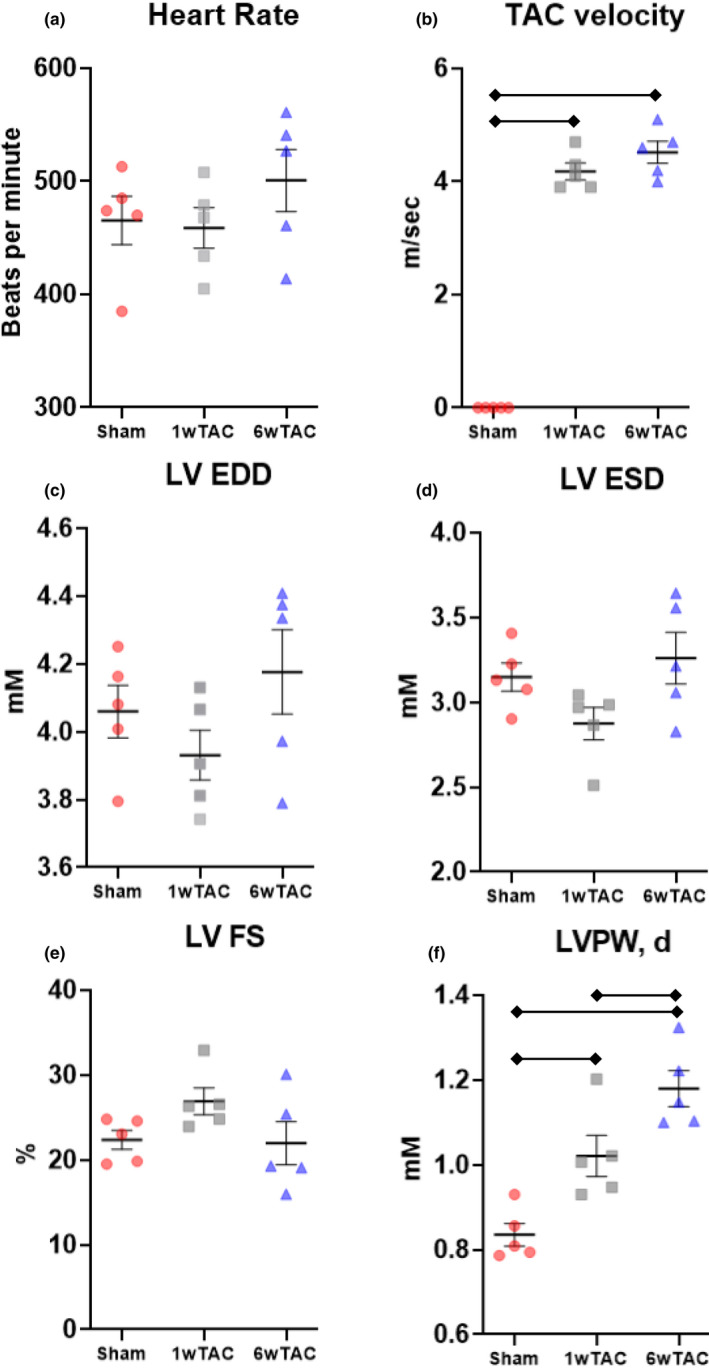
Echocardiographic measurements in male mice. Bars indicate mean ± standard error of the mean. Brackets designate *p *< 0.05 between the indicated groups. TAC, transverse aortic constriction; LV EDD, left ventricular end‐diastolic diameter; LV ESD, left ventricular end‐systolic diameter; LV FS, left ventricular fractional shortening; LVPW,d, left ventricular posterior wall thickness in diastole. *N *= 5 for all groups

**FIGURE 3:**
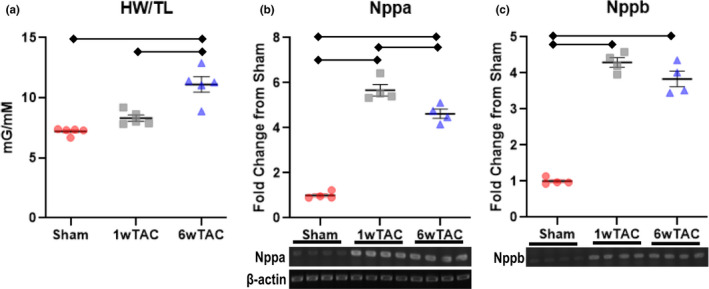
Heart weigh normalized to tibial length (HW/TL) and mRNA transcript levels for natriuretic peptide A (Nppa) and natriuretic peptide B (Nppb) in males. For Nppa and Nppb, beta‐actin transcript levels were used as a sample control and shown in B. Nppa and Nppb transcript levels were normalized to their appropriate beta‐actin sample control and then reported relative to the average transcript level of the Sham group. Bars indicate mean ± standard error of the mean. Brackets designate *p* < 0.05 between the indicated groups. *N *= 5 per group for HW/TL and *n *= 4 per group for Nppa and Nppb

We performed echocardiograms to assess cardiac parameters and the degree of aortic constriction. In male mice (Figure 2), cardiac parameters were similar among the groups except left ventricular posterior wall thickness in diastole increased in both TAC groups compared to sham with a further increase from 1wTAC to 6wTAC (Figure 2f). The velocity of flow across the aortic constriction site (TAC velocity) was similar between TAC groups (Figure 2b) with an estimated a peak instantaneous pressure gradient of 70 ± 5 mmHg in 1wTAC and 82 ± 7 in 6wTAC. We assessed hypertrophic growth by measuring heart weight normalized to tibial length. Normalized heart weight was similar between sham and 1wTAC (*p *= 0.08), but higher in 6wTAC versus the other groups (Figure 3a). We also assessed mRNA transcript levels of Nppa and Nppb (Figure 3b and 3c), which are frequently used as markers for hypertrophy and/or heart failure. Nppa and Nppb were significantly higher in both 1wTAC and 6wTAC compared to sham, with a further increase in 1wTAC versus 6wTAC for Nppa only. Our results show active hypertrophic growth in 1wTAC with relatively preserved systolic function between 1wTAC and 6wTAC.

**FIGURE 4:**
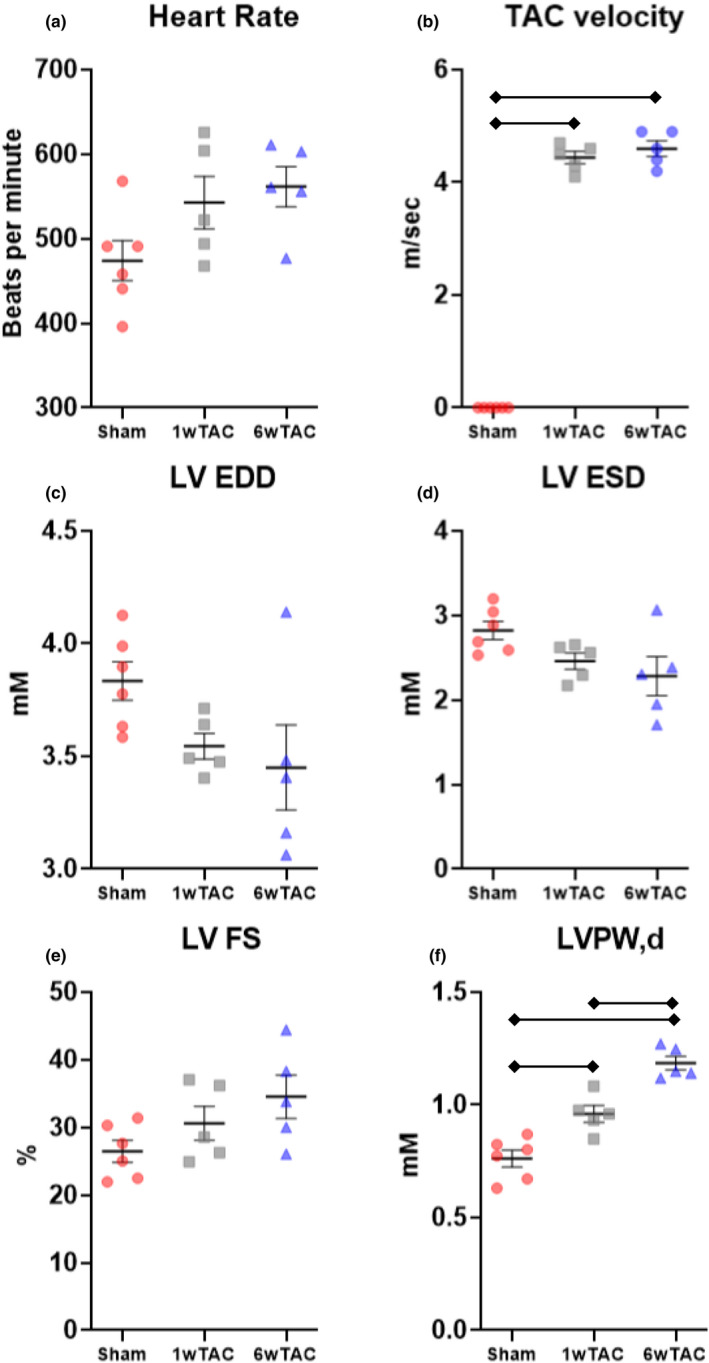
Echocardiographic measurements in female mice. Bars indicate mean ± standard error of the mean. Brackets designate *p* < 0.05 between the indicated groups. TAC, transverse aortic constriction; LV EDD, left ventricular end‐diastolic diameter; LV ESD, left ventricular end‐systolic diameter; LV FS, left ventricular fractional shortening; LVPW,d, left ventricular posterior wall thickness in diastole. *N *= 5 for 1wTAC and 6wTAC, *N *= 6 for sham

**FIGURE 5:**
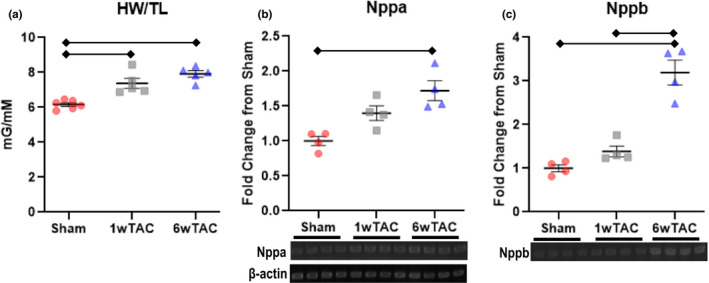
Heart weigh normalized to tibial length (HW/TL) and mRNA transcript levels for natriuretic peptide A (Nppa) and natriuretic peptide B (Nppb) in female mice. For Nppa and Nppb, beta‐actin transcript levels were used as a sample control and shown in B. Nppa and Nppb transcript levels were normalized to their appropriate beta‐actin sample control and then reported relative to the average transcript level of the sham group. Bars indicate mean ± standard error of the mean. Brackets designate *p* < 0.05 between the indicated groups. For HW/TL, *n *= 6 for sham, and *n *= 5 for 1wTAC and 6wTAC. For Nppa and Nppb, *n *= 4 per group

Female mice also had similar echocardiographic parameters among the groups (Figure 4) except for increased left ventricular posterior wall thickness in diastole in both TAC groups compared to sham and between 1wTAC and 6wTAC (Figure 4f). The velocity of flow across the aortic constriction site (TAC velocity) was similar between female TAC groups (Figure 4b) with an estimated a peak instantaneous pressure gradient of 79 ± 4 mmHg in 1wTAC and 85 ± 5 mmHg in 6wTAC. Normalized heart weight was higher in both 1wTAC and 6wTAC compared to sham, but unchanged between 1wTAC and 6wTAC (Figure 5a). Nppa transcript levels (Figure 5b) were greater in 6wTAC versus sham with a nonsignificant increase in 1wTAC versus sham (*p *= 0.068). Nppb transcript levels increased in 6wTAC versus both 1wTAC and sham (Figure 5c). Overall, hypertrophic growth as assessed by heart mass appears to be nearing completion around 1 week after TAC in the female mice with relatively stable cardiac systolic function until 6wTAC in our model. The increase in Nppb for 6wTAC versus both 1wTAC and sham may indicate the beginning of a decline in cardiac systolic function.

### 3.2 Temporal regulation of *O*‐GlcNAc levels after TAC

**FIGURE 6:**
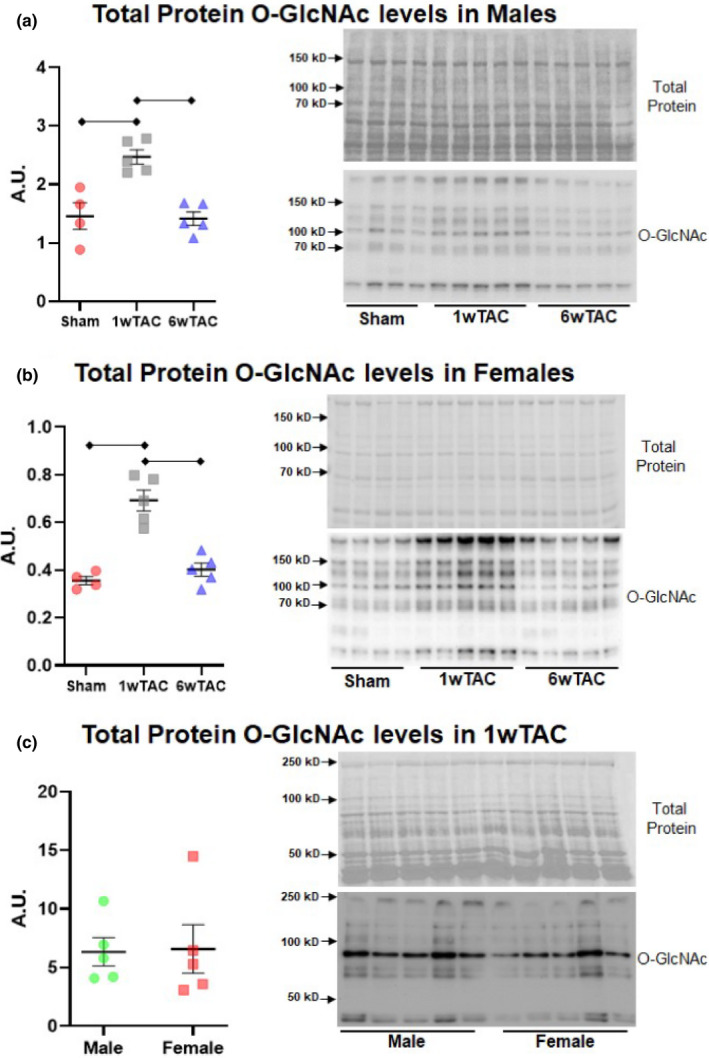
Total protein *O*‐GlcNAc levels in males and females. Immunoblots are shown for total protein *O*‐GlcNAc levels normalized to protein staining in males (a), females (b), and males and females at 1wTAC (c). Bars indicate mean ± standard error of the mean. Brackets designate *p* < 0.05 between the indicated groups. A.U.; arbitrary units. *N *= 4 for sham, and *N *= 5 for 1wTAC and 6wTAC (both sexes)

We used western blots to determine total protein *O*‐GlcNAc levels as well as regulation of its principal enzymes during TAC. In both males and females, myocardial total protein *O*‐GlcNAc levels rose at 1wTAC versus sham with a subsequent drop in 6wTAC (Figure 6a and 6b, Supplemental Figure S1A and S1B). Total protein *O*‐GlcNAc levels also rose to similar levels in 1wTAC for males versus females (Figure 6c).

**FIGURE 7:**
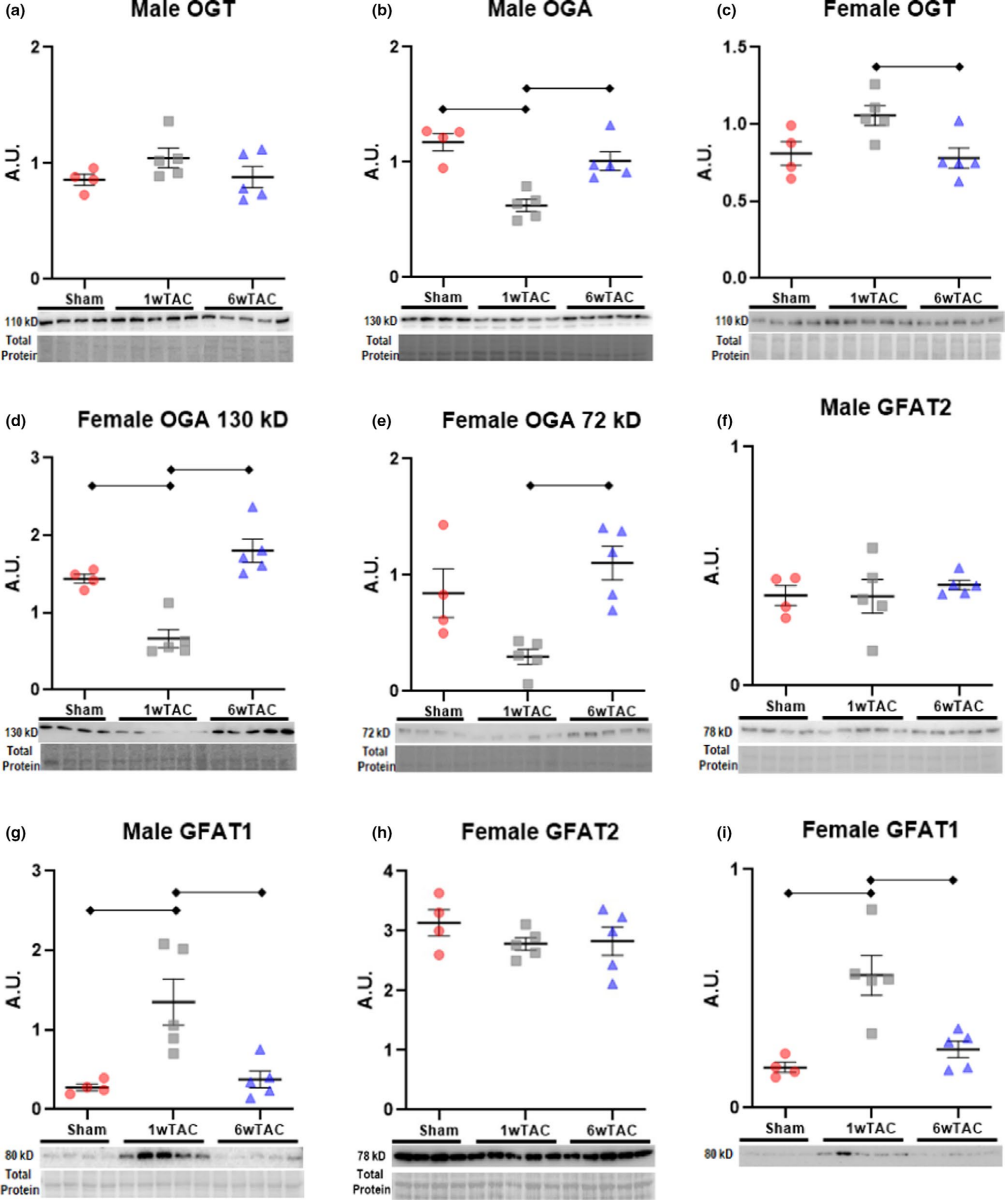
Protein levels of key enzymes for protein O‐GlcNAcylation and the hexosamine biosynthesis pathway. Immunoblots normalized to protein staining in male and female mice for OGT, OGA, GFAT2, and GFAT1. Note, H and I were performed on the same membrane after stripping and, therefore, share a common protein straining region for normalization shown in H. Bars indicate mean ± standard error of the mean. Brackets designate *p* < 0.05 between the indicated groups. A.U.; arbitrary units. *N *= 4 for sham, and *N *= 5 for 1wTAC, and 6wTAC (both sexes)

We next evaluated the primary enzymes regulating *O*‐GlcNAc levels (Figure 7). OGT attaches *O*‐GlcNAc to proteins whereas OGA performs the removal. In males, OGT levels remained unchanged among the groups (Figure 7a); however, OGA levels dropped in 1wTAC versus both sham and 6wTAC (Figure 7b). In females, OGT was higher in 1wTAC compared to the other groups, but this difference reached significance only versus 6wTAC (*p *= 0.03) and not sham (*p *= 0.07) (Figure 7c). Female OGA significantly dropped in 1wTAC versus both sham and 6wTAC for the isoform at 130 kD, which was the same isoform identified in males (Figure 7d). The 130‐kD isoform is principally found in the cytoplasm. Females also had an additional OGA isoform identified at 72 kD which is typically located in the nucleus. The 72‐kD OGA was significantly lower 1wTAC versus 6wTAC with a nonsignificant decrease in 1wTAC versus sham (*p *= 0.06) (Figure 7e). GFAT is the rate‐limiting enzyme of the HBP and has two isoforms (GFAT1 and GFAT2) with GFAT2 as the primary myocardial variant. In both males and females, GFAT2 remained unchanged after TAC (Figure 7f and 7h). However, we found significantly higher GFAT1 protein levels in 1wTAC versus the other groups for both males and females (Figure 7g and 7i).

**FIGURE 8:**
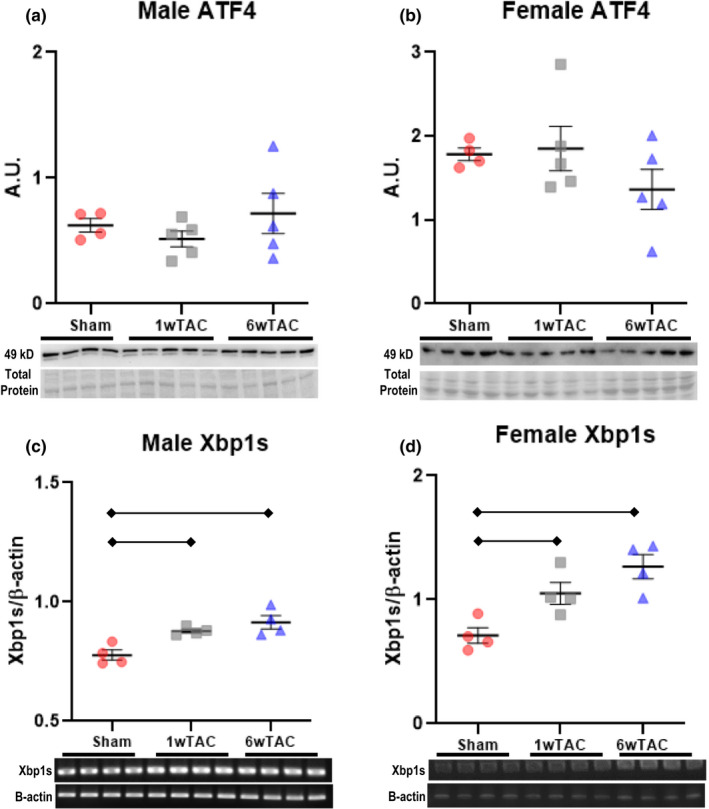
Activating transcription factor 4 (ATF4) protein levels and X‐box‐binding protein 1 splice variant (XBP1s) transcript levels in males and females. ATF4 immunoblots normalized to protein staining are shown for males (a) and females (b). XBP1s mRNA transcript levels normalized to beta‐actin is shown for males (c) and females (d). Bars indicate mean ± standard error of the mean. Brackets designate *p* < 0.05 between the indicated groups. A.U.; arbitrary units. For a and b, *n *= 4 for sham, *n *= 5 for 1wTAC and 6wTAC. For c and d, *n *= 4 per group

Prior work found myocardial GFAT1 induction by XBP1s or ATF4 (Nabeebaccus et al., 2017; Wang et al., 2014), both of which are part of the unfolded protein response (UPR) triggered by accumulation of misfolded proteins in the endoplasmic reticulum. Since active hypertrophic growth could theoretically trigger the UPR, we evaluated whether ATF4 or Xbp1s could promote myocardial GFAT1 induction in our model. ATF4 protein levels remained unchanged after TAC (Figure 8a and 8b). Like previous studies, we used RT‐PCR to measure Xbp1s expression (Wang et al., 2014). Both TAC groups had higher Xbp1s transcript levels than sham, but there was no difference between 1wTAC and 6wTAC (Figure 8c and 8d). Thus, there was no correlation between GFAT1 levels and ATF4 or Xbp1s.

### 3.3 Augmenting *O*‐GlcNAc levels during prolonged pressure‐overload (established hypertrophy)

**FIGURE 9:**
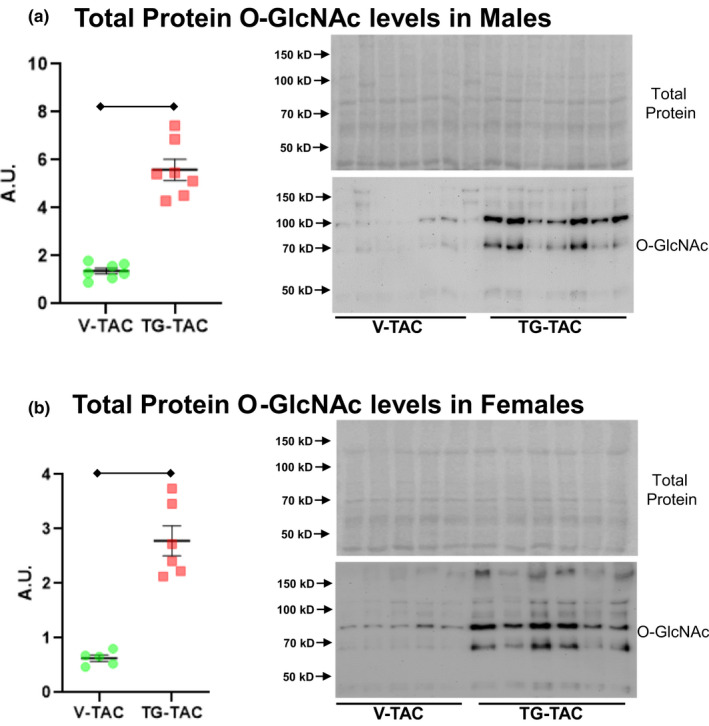
Total protein *O*‐GlcNAc levels during established pressure‐overload hypertrophy with injections of thiamet‐g (TG‐TAC) or vehicle (V‐TAC). Immunoblots are shown for total protein *O*‐GlcNAc levels normalized to protein staining in (a) males and (b) females. Bars indicate mean ± standard error of the mean. Brackets designate *p* < 0.05 between the indicated groups. A.U.; arbitrary units. *N *= 7 for male V‐TAC, N = 7 for male TG‐TAC, *N *= 5 for female V‐TAC and *N *= 6 for female TG‐TAC

**FIGURE 10:**
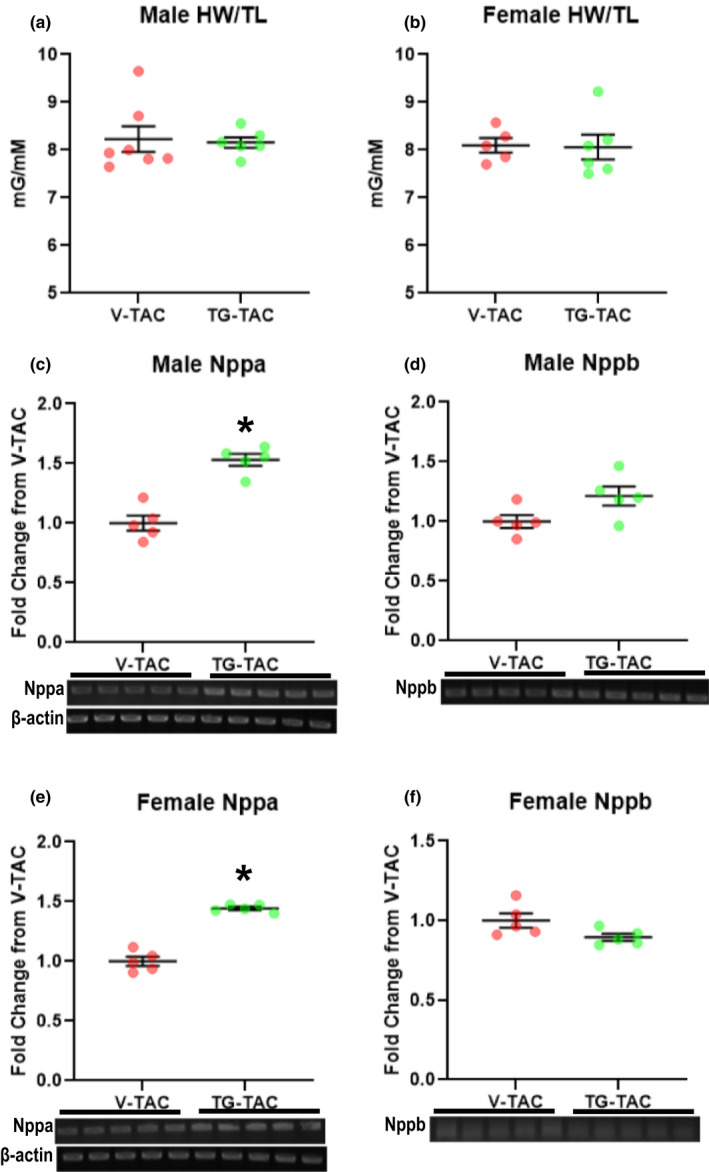
Heart weigh normalized to tibial length (HW/TL), and mRNA transcript levels for natriuretic peptide A (Nppa) and natriuretic peptide B (Nppb) in male and female mice treated with thiamet‐g (TG‐TAC) or vehicle (V‐TAC) during established hypertrophy. HW/TL shown for males (a) and females (b). Nppa transcript levels shown for males (c) and females (e). Nppb transcript levels shown for males (d) and females (f). For Nppa and Nppb, beta‐actin transcript levels were used as a sample control and shown in c for males and e for females. Nppa and Nppb transcript levels were normalized to their appropriate beta‐actin sample control and then reported relative to the average transcript level of the appropriate V‐TAC group. Bars indicate mean ± standard error of the mean. Brackets designate *p* < 0.05 between the indicated groups. For HW/TL, *n *= 7 for male V‐TAC, *n *= 7 for male TG‐TAC, *n *= 5 for female V‐TAC and *n *= 6 for female TG‐TAC. For Nppa and Nppa, *n *= 5 for all groups

Cardiac specific knock‐out of GFAT1 prior to TAC lowers *O*‐GlcNAc levels while reducing cardiac hypertrophy (Tran et al., 2020). Thus, we questioned whether the fall in total protein O‐GlcNAcylation in 6wTAC inhibits ongoing hypertrophic growth. We tested this possibility by injecting the OGA inhibitor TG to increase protein O‐GlcNAcylation during established hypertrophy (TG‐TAC). Control mice underwent TAC but received vehicle injections (V‐TAC). TG significantly increased protein *O*‐GlcNAc levels by approximately 4.3‐fold in males and 4.5‐fold in females (Figure 9a and 9b). However, normalized heart mass did not change with the higher *O*‐GlcNAc levels during established hypertrophy for either gender (Figure 10a and 10b). There was a small, but significant increase in Nppa transcript levels in TG‐TAC for both genders (Figure 10c and 10e). Nppb transcript levels had a nonsignificant increase in TG‐TAC versus V‐TAC for males (*p *= 0.057, Figure 10d), whereas there was a nonsignificant decrease in TG‐TAC versus V‐TAC for females (*p *= 0.069, Figure 10f).

**FIGURE 11:**
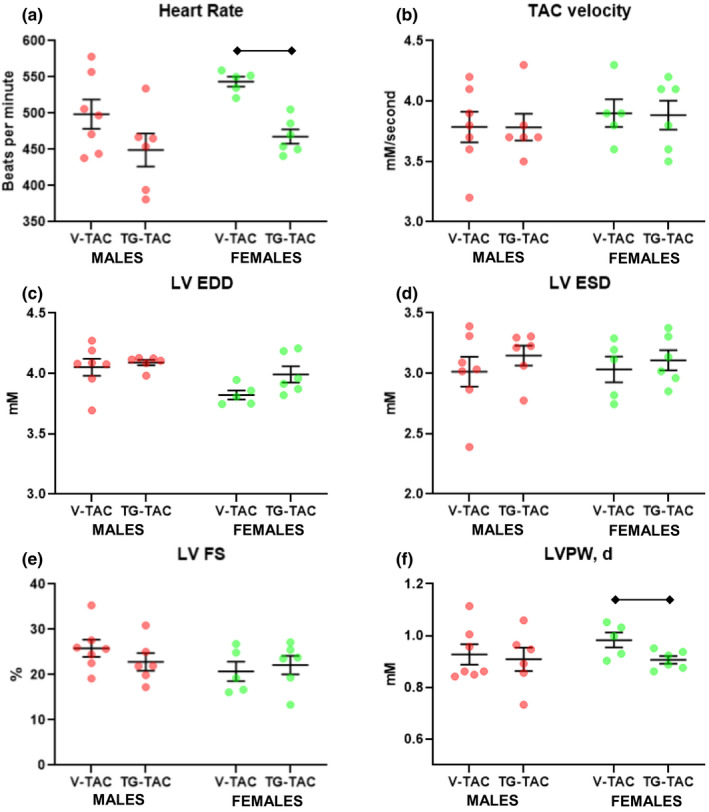
Echocardiographic measurements in male and female mice during established hypertrophy treated with thiamet‐g (TG‐TAC) or vehicle (V‐TAC). Bars indicate mean ± standard error of the mean. Brackets designate *p* < 0.05 between the indicated groups. TAC, transverse aortic constriction; LV EDD, left ventricular end‐diastolic diameter; LV ESD, left ventricular end‐systolic diameter; LV FS, left ventricular fractional shortening; LVPW,d, left ventricular posterior wall thickness in diastole. *N *= 7 for male V‐TAC, *N *= 7 for male TG‐TAC, *N *= 5 for female V‐TAC and *N *= 6 for female TG‐TAC

Sustained protein *O*‐GlcNAc elevations cause cardiac dysfunction in diabetic cardiomyopathy (Clark et al., 2003; Fricovsky et al., 2012; Hu et al., 2005; Yokoe et al., 2010), but it is not clear whether this also occurs with TAC. Accordingly, we also evaluated cardiac systolic function via echocardiograms. Prior to starting the injections; cardiac systolic function, parameters of left ventricular size, and velocity across the aortic constriction site were similar between the TG‐TAC versus V‐TAC in both males and females (data not shown). There were no differences in cardiac parameters measured by echocardiogram on the day prior to tissue collection between male TG‐TAC and V‐TAC (Figure 11). In female mice, TG‐TAC had slower heart rates (Figure 11a) and decreased left ventricular posterior wall thickness in diastole compared with V‐TAC (Figure 11f) with a nonsignificant tread toward increased left ventricular end diastolic dimension (*p*=0.06, Figure 11d). Core body temperature did not affect the female heart rate as both groups were maintained around 37.5℃.

### 3.4 Effect of Protein O‐GlcNAcylation on Xbp1s during established hypertrophy

**FIGURE 12:**
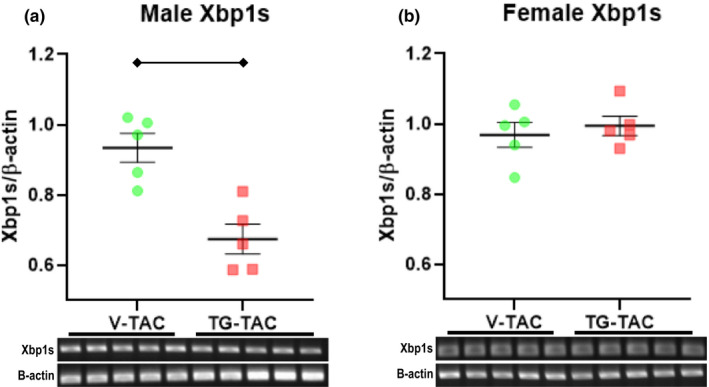
X‐box‐binding protein 1 splice variant (XBP1s) transcript levels in male (a) and female mice (b) during established hypertrophy treated with thiamet‐g (TG‐TAC) or vehicle (V‐TAC). XBP1s mRNA transcript levels normalized to beta‐actin are shown for males (a) and females (b). Bars indicate mean ± standard error of the mean. Brackets designate *p* < 0.05 between the indicated groups. *N *= 5 for all groups

ATF4 and Xbp1s did not appear to regulate GFAT1 and protein *O*‐GlcNAc levels during rapid hypertrophic growth. Since O‐GlcNAcylation can reciprocally affect protein quality control (Denzel et al., 2014), we assessed whether augmenting *O*‐GlcNAc levels lowered Xbp1s transcript levels during established hypertrophy. We focused on Xbp1s since transcript levels remained elevated at 6wTAC (shown previously in Figure 8c and 8d). Xbp1s fell slightly in TG‐TAC versus V‐TAC in male mice (Figure 12a); however, there was no difference in female mice (Figure 12b).

## 4 DISCUSSION

Increased protein O‐GlcNAcylation is well‐recognized during cardiac hypertrophy. Prior studies primarily evaluated O‐GlcNAcylation at a single time during active hypertrophic growth in the early weeks after TAC but neglected comparisons to established hypertrophy. We tested the hypothesis that key regulatory enzymes are induced to increase protein O‐GlcNAcylation primarily during active myocardial hypertrophic growth. We show for the first time temporal changes in protein *O*‐GlcNAc levels and its regulatory enzymes GFAT1 and OGA during POH. Total protein *O*‐GlcNAc levels increase in 1wTAC with a return to sham levels at 6wTAC. Thus, we confirmed augmentation of protein O‐GlcNAcylation during rapid hypertrophic growth early after TAC but not with established hypertrophy during prolonged TAC. Notably, both genders demonstrated a similar pattern in protein *O*‐GlcNAc levels during POH. Differing from other cardiac pathologies, GFAT1 induction does not appear to be from the UPR mechanisms AFT4 and Xbp1s.

We further tested whether downregulation of protein O‐GlcNAcylation inhibits ongoing hypertrophic growth with prolonged pressure‐overload during established hypertrophy. We gave the OGA inhibitor TG starting 4 weeks after TAC surgery to increased protein *O*‐GlcNAc levels. Although cardiac size was unchanged in these mice, increased O‐GlcNAcylation appeared to reduce unfolded protein stress in males suggesting a potential beneficial action.

### 4.1 Regulation of protein *O*‐GlcNAc levels during POH

Our results add important insights into the regulation of protein O‐GlcNAcylation during POH. Given the connection between glycolysis and the HBP, it is widely accepted that increased flux of glucose through the initial glycolysis steps (increased glucose availability) is the key determinant of HBP flux and *O*‐GlcNAc levels (Fliegner et al., 2010). Consequently, higher *O*‐GlcNAc levels in cardiac hypertrophy are commonly attributed to increased glycolysis (Kreppel & Hart, 1999; Taegtmeyer et al., 2016; Watson et al., 2010; Weinberg et al., 1999). However, our results suggest that protein *O*‐GlcNAc levels do not simply reflect glycolytic rates during hypertrophy. Furthermore, we provide additional confirmation on the importance of non‐nutrient regulation of protein *O*‐GlcNAc levels. We found that increased protein *O*‐GlcNAc levels correlate with GFAT1 induction and lower OGA levels after TAC. Female mice also demonstrated greater OGT levels during early hypertrophy. We speculate that GFAT1 induction increases HBP flux making more UDP‐GlcNAc available for OGT. Additionally, lower OGA levels theoretically increase overall *O*‐GlcNAc levels by reducing removal of this protein PTM. The totality of these enzymatic changes would increase protein *O*‐GlcNAc levels during early pressure‐overload.

Tran et al. similarly found increased GFAT1 protein levels with TAC, but they did not assess OGT or OGA (Tran et al., 2020). Cardiac‐specific inducible GFAT1 knockout (GFAT1KO) prior to TAC reduced protein *O*‐GlcNAc levels and cardiomyocyte hypertrophy 3 weeks post‐surgery. Tran et al. did not directly measure HBP flux or GFAT enzyme activity, so we do not know the specific effect of GFAT1 on HBP flux during hypertrophy. Paradoxically, transgenic GFAT1 overexpression did not increase protein *O*‐GlcNAc levels in sham hearts (Tran et al., 2020). Thus, we speculate that GFAT1 induction requires additional factors to increase HBP flux and/or *O*‐GlcNAc levels during early hypertrophy. Our current results suggest OGA repression may also be necessary to augment protein *O*‐GlcNAc levels during early hypertrophy. Changes in other enzymes and/or nutrient availability (such as glucose or glutamine) may also be required to alter *O*‐GlcNAc levels in conjunction with GFAT1 induction during POH. Further studies are necessary to clarify regulation of HBP flux and protein *O*‐GlcNAc kinetics during hypertrophy including experiments directly measuring HBP flux.

We were unable to determine the mechanisms for GFAT1 induction or OGA repression. Based on previous myocardial studies, we focused on the UPR pathways Xbp1s and ATF4 as candidate mechanisms promoting GFAT1 induction. Nabeebaccus et al. created pressure overload hypertrophy via suprarenal aortic constriction and found GFAT1 induction via a NADPH oxidase‐4‐ATF4‐dependent mechanism (Nabeebaccus et al., 2017). In our study, however, ATF4 protein levels remained similar among sham, 1wTAC, and 6wTAC. Wang et al found Xbp1s promoted transcription of HBP enzymes including GFAT1 during cardiac ischemia/reperfusion in mice (Wang et al., 2014). We found that Xbp1s transcript levels increased equally over sham for both early and established hypertrophy. Thus, neither ATF4 protein nor Xbp1s transcript levels specifically correlate with the dynamic changes in GFAT1 levels after TAC. Our finding suggests GFAT1 is regulated by multiple mechanisms that differ based upon the cardiac pathology.

Little is known about regulation of myocardial OGA levels. In order to maintain consistent overall protein *O*‐GlcNAc levels, OGA and OGT protein levels frequently increase or decrease together. However, we found that OGA levels dropped without a concordant fall in OGT. An earlier study showed that miR‐539 specifically represses OGA without affecting OGT (Muthusamy et al., 2014). In a mouse model of myocardial infarction, increased *O*‐GlcNAc levels with reduced OGA correlated to miR‐539 expression. Follow‐up cell culture experiments demonstrated miR‐539 specifically reduced OGA protein levels and increased protein O‐GlcNAcylation. Future studies should determine whether miR‐539 expression changes between early and established POH.

We also made the novel finding that total protein *O*‐GlcNAc levels return to baseline (Sham) levels with established hypertrophy. We must note important caveats with this result. First, we report only total protein levels. Thus, we do not know whether O‐GlcNAcylation differs for select proteins during established hypertrophy. Identification of *O*‐GlcNAcylated proteins and modification sites is difficult with standard mass spectroscopy methods and conditions due to the fragility of the *O*‐GlcNAc bond. Future studies should employ new mass spectroscopy methods to identify specific *O*‐GlcNAcylated proteins during cardiac hypertrophy as well as determine whether temporal differences occur in *O*‐GlcNAc protein specificity. Second, limited studies in humans demonstrate sustained elevations in total protein *O*‐GlcNAc levels with chronic symptomatic aortic stenosis (Lunde et al., 2012). These human samples were obtained from patients undergoing aortic valve replacement surgery indicating severe disease compared to the relatively preserved cardiac systolic function in our established hypertrophy mice (6wTAC). Thus, we speculate severe uncompensated heart failure may be an additional trigger for augmenting protein *O*‐GlcNAc levels. On the other hand, *O*‐GlcNAc regulation may differ between humans and mice during hypertrophy.

### 4.2 Gender and protein O‐GlcNAcylation during POH

Males have primarily been used in the previous studies on protein O‐GlcNAcylation during POH (Dassanayaka et al., 2017; Zhu et al., 2019). To our knowledge, this is the first study specifically evaluating gender differences in *O*‐GlcNAc and regulatory enzymes with TAC. It is important to assess gender differences because female gender affects both cardiac metabolism and hypertrophic growth. We found that female mice had lower normalized heart weights even with comparably increased afterload from TAC. Even so, males and females had a similar temporal pattern of protein *O*‐GlcNAc levels after TAC, as well as, changes in GFAT1 and OGA. We detected the 72‐kD OGA isoform in females only. We are not aware of gender differences in OGA isoforms in the heart or other organs, but this should be explored in future studies. OGT protein levels increased in early hypertrophy for females only. OGT resides on the X‐chromosome, which may underlie the gender differences in expression of this protein. Although overall O‐GlcNAc levels appears similar, future studies should evaluate whether O‐GlcNAc protein specificity or functional effects differ between the genders during cardiac hypertrophy. For example, we found increased O‐GlcNAcylation from TG during established hypertrophy reduced Xbp1s transcript levels in males only (discussed later).

### 4.3 Augmenting *O*‐GlcNAc levels during established hypertrophy

*O*‐GlcNAc levels returned to baseline (Sham) levels with established hypertrophy. Thus, we wondered whether the decline in protein *O*‐GlcNAc levels during established hypertrophy arrests hypertrophic growth. Some, but not all, studies suggest *O*‐GlcNAc promotes cardiac hypertrophic growth. As noted, cardiac specific GFAT1 knock‐out reduced protein *O*‐GlcNAc levels while reducing cardiac hypertrophy with TAC (Tran et al., 2020). Similarly, Gelinas and Mailleux et al. showed that AMPK inhibits cardiac hypertrophy by reducing O‐GlcNAcylation both *in vitro* and *in vivo* (Gélinas et al., 2018). Paradoxically, we previously found profoundly reducing protein *O*‐GlcNAc levels via OGT knock‐out had no effect on hypertrophic growth initially after TAC, but increased cardiomyocyte size and left ventricular dilation during established hypertrophy (Zhu et al., 2019). In the current study, we gave TG to augment protein *O*‐GlcNAc levels during established hypertrophy. TG caused a small but significant increase in Nppa transcript levels; however, cardiac mass remained unchanged even with the higher *O*‐GlcNAc levels. Therefore, our results suggest increased protein O‐GlcNAcylation principally affects hypertrophic growth early after TAC. We propose three potential mechanisms for *O*‐GlcNAc's inconsistent effects on hypertrophy. First, changes in the relationship between *O*‐GlcNAc and hypertrophic growth may be due to variances in the specificity of O‐GlcNAcylated proteins between early versus established hypertrophy. Second, *O*‐GlcNAc may require co‐activation with additional factor(s) to promote hypertrophic growth. As one example, *O*‐GlcNAc and protein phosphorylation demonstrate extensive crosstalk (Butkinaree et al., 2010). The interaction between *O*‐GlcNAc and phosphorylation may vary over time on specific proteins leading to differential effects on hypertrophic growth. Third, thiamet‐g increases protein *O*‐GlcNAc levels systemically. Thus, it is possible that systemic O‐GlcNAcylation counteracts *O*‐GlcNAc's cardiac specific effects. Future studies should evaluate potential mechanisms causing *O*‐GlcNAc's inconsistent effects on hypertrophic growth.

We also evaluated whether TG affects cardiac systolic function or unfolded protein stress during established hypertrophy. The functional effects of protein O‐GlcNAcylation are frequently thought to be dichotomous with acute increases promoting adaptation to physiological stressors, whereas prolonged elevations are hypothesized to cause disease pathology (for review see Yang & Qian, 2017). Thus, it is notable that cardiac systolic function, assessed by echocardiogram, was similar between TG‐TAC and V‐TAC for both sexes. In females, we speculate that the variation in ventricular size and posterior wall thickness in TG‐TAC is secondary to a slower heart rate compared to V‐TAC. Male TG‐TAC mice had lower Xbp1s transcript levels suggesting that elevating *O*‐GlcNAc levels beneficially reduces overall unfolded protein stress during established hypertrophy. Conceivably, long‐term reductions in unfolded protein stress by O‐GlcNAcylation could prevent compensated hypertrophy from progressing to heart failure but further studies are required to test this possibility. Future studies should also evaluate the mechanisms underlying the gender differences in *O*‐GlcNAc's effect on Xbp1s.

### 4.4 Limitations

We speculate that protein levels of OGT, OGA, and GFAT1 reflect activity of these enzymes. However, further studies are needed to measure the effect of these changes on actual HBP flux and/or enzyme activity. Second, additional factors beyond OGT, OGA, and GFAT1 are potentially required to regulate HBP flux and protein *O*‐GlcNAc levels during POH. For example, although we previously found that glycolic rate does not affect HBP flux in non‐hypertrophied hearts; we do not know whether hypertrophy and/or GFAT1 induction reset the relationship between HBP flux and glucose availability. Third, we cannot exclude that Xbp1s partially regulates GFAT1 induction without specifically inhibiting Xbp1s gene expression with transgenic mice. Finally, we did not measure cardiomyocyte cross‐sectional area in V‐TAC versus TG‐TAC, so we cannot exclude the possibility of a small difference in cardiomyocyte size between these groups.

## 5 CONCLUSION

Protein *O*‐GlcNAc levels are temporally regulated after TAC with elevated levels occurring during rapid hypertrophic growth initially after aortic constriction in both males and females. Increased *O*‐GlcNAc levels appear at least partially regulated by changes in the enzymes GFAT1, OGA, and OGT (females only). Pharmacologically increasing *O*‐GlcNAc levels during established hypertrophy did not affect cardiac mass or systolic function but may beneficially reduce unfolded protein stress.

## CONFLICTS OF INTEREST

The authors have no conflicts of interest to disclosure.

## AUTHOR CONTRIBUTIONS

AO had the idea for the study. AO and WZ designed the study. AO, WZ, DL conducted the experiments, gathered and analyzed the data, and interpreted the results. AO wrote the manuscript. AO, WZ, and DL proofread and approved the final version of the manuscript.

## Supporting information



Fig S1

Fig S2
