# Supplementary material for: Temporal regulation of protein O‐GlcNAc levels during pressure‐overload cardiac hypertrophy
Source: Physiol Rep. 2021 Aug 2;9(15):e14965. doi: 10.14814/phy2.14965 (PMC8326887; doi:10.14814/phy2.14965)

A. Total protein O-GlcNAc levels using CTD110.6 antibody in males

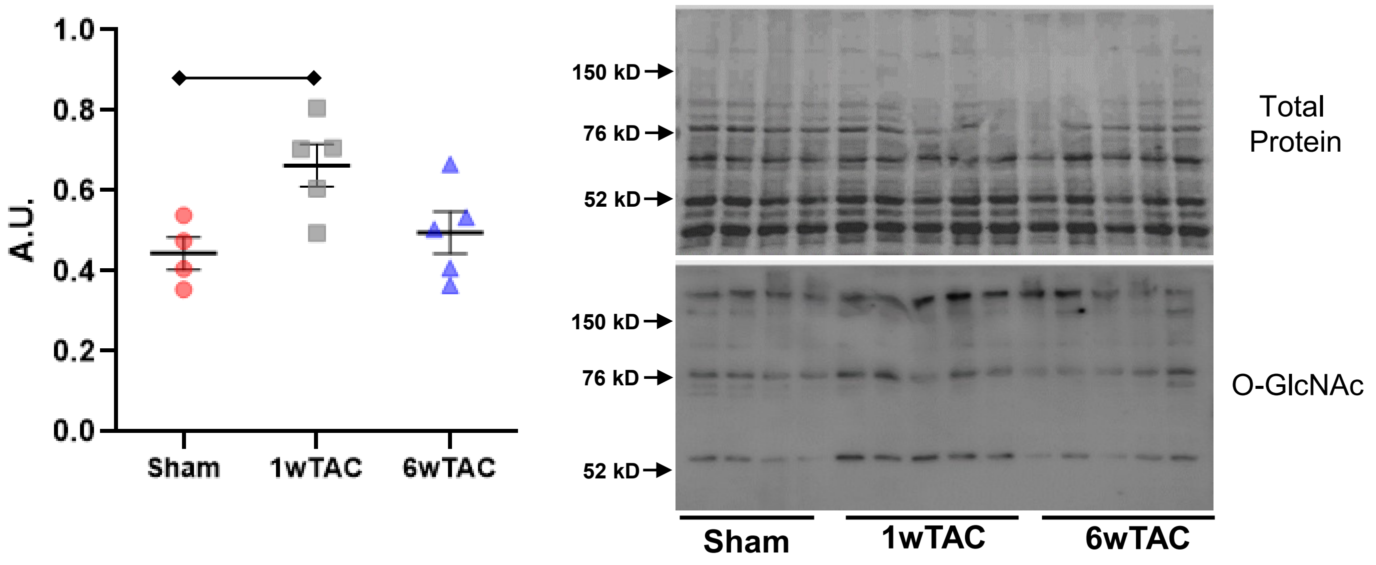

B. Total protein O-GlcNAc levels using CTD110.6 antibody in females

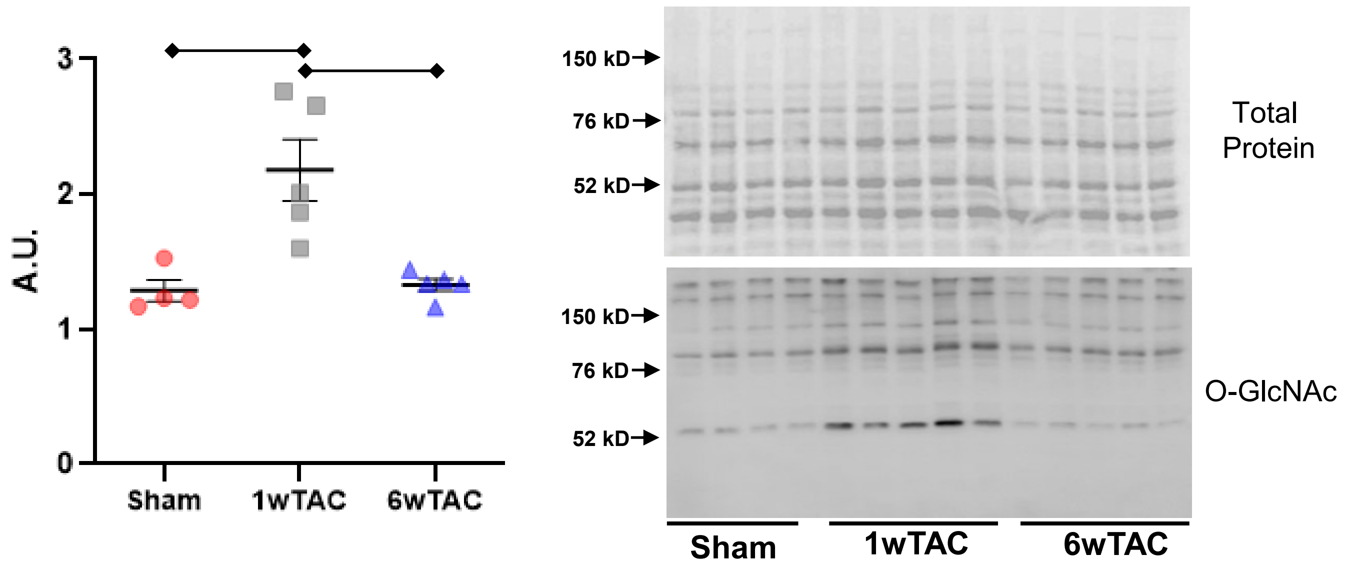

Supplement: Supplementary file 1 — Fig S1 [file PHY2-9-e14965-s001.pdf]
