# Supplementary material for: Temporal regulation of protein O‐GlcNAc levels during pressure‐overload cardiac hypertrophy
Source: Physiol Rep. 2021 Aug 2;9(15):e14965. doi: 10.14814/phy2.14965 (PMC8326887; doi:10.14814/phy2.14965)

Figure 6A.

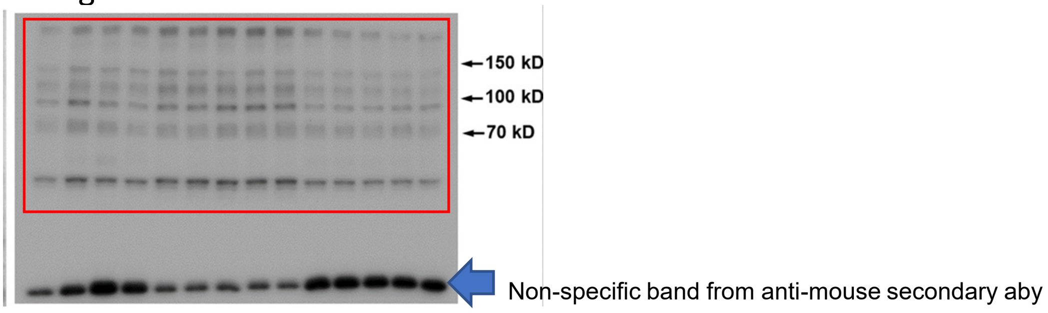

Figure 6B.

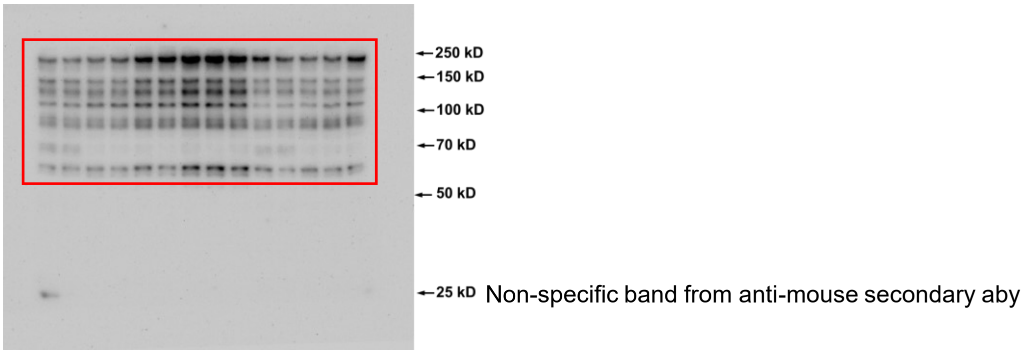

Figure 6C.

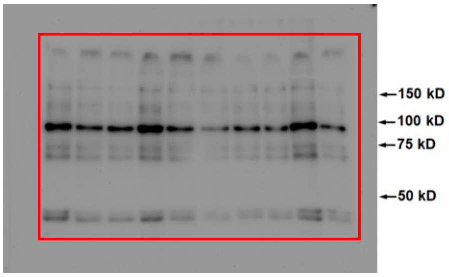

Figure 7A.

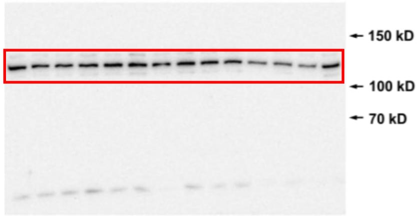

Figure 7B.

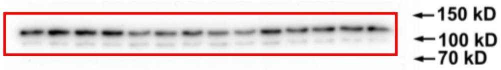

Figure 7C.

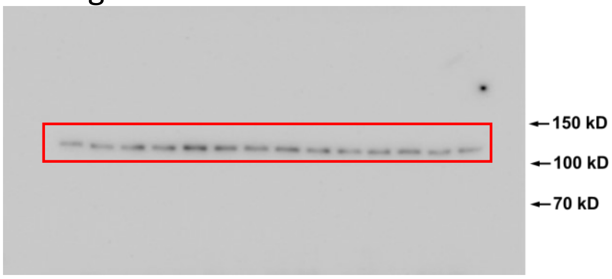

Figure 7D and 7E.

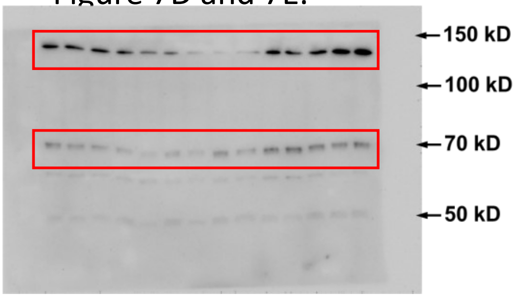

Figure 7F

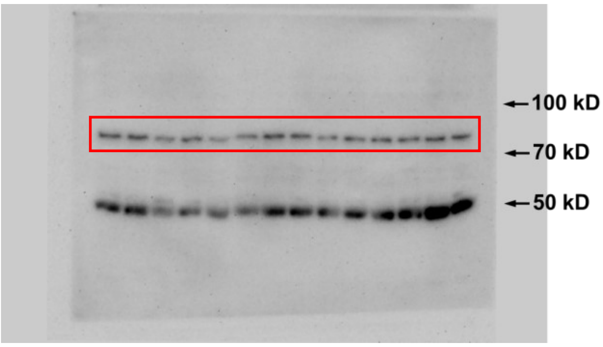

Figure 7G

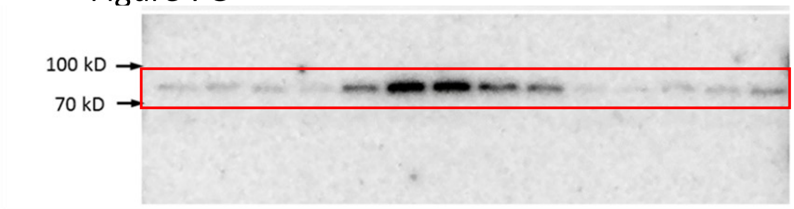

Figure 7H

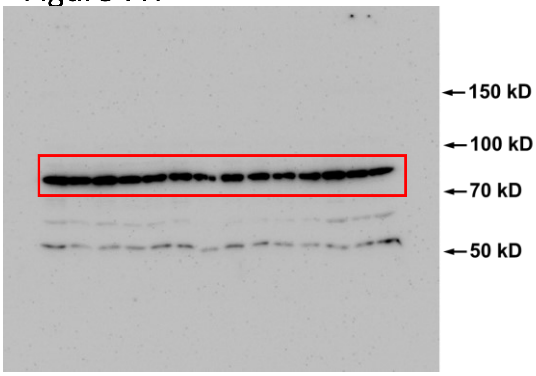

Figure 7I

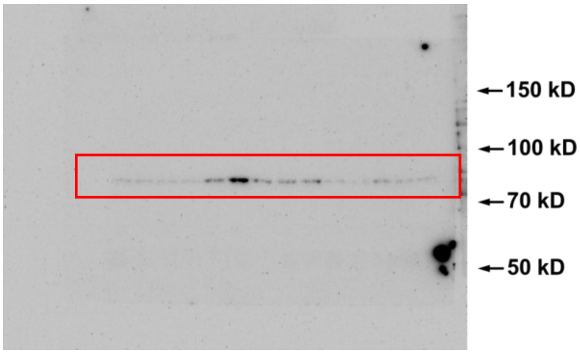

Figure 8A

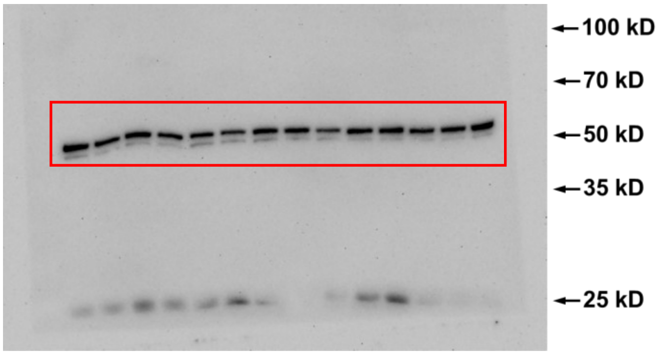

Figure 8B

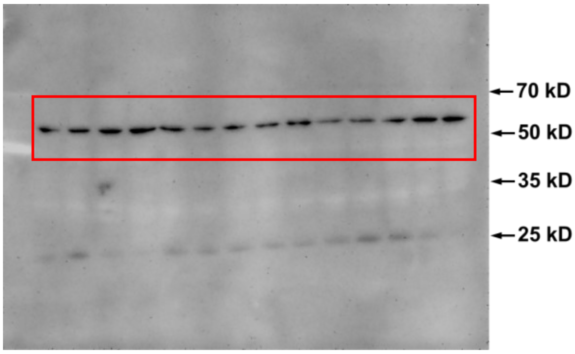

Figure 9A

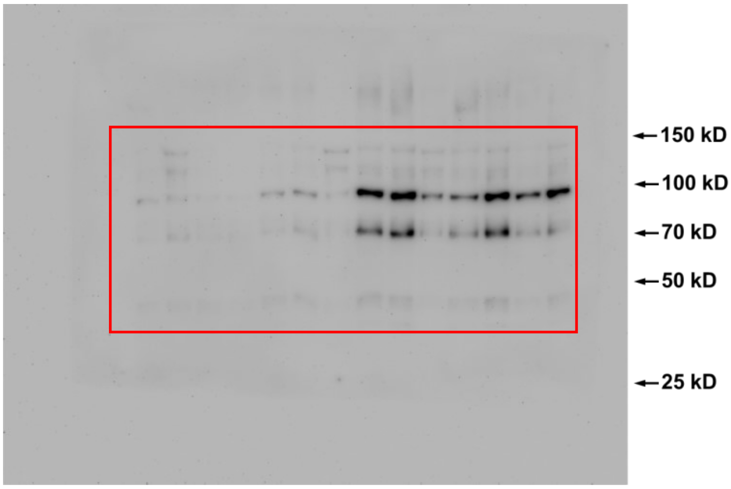

Figure 9B

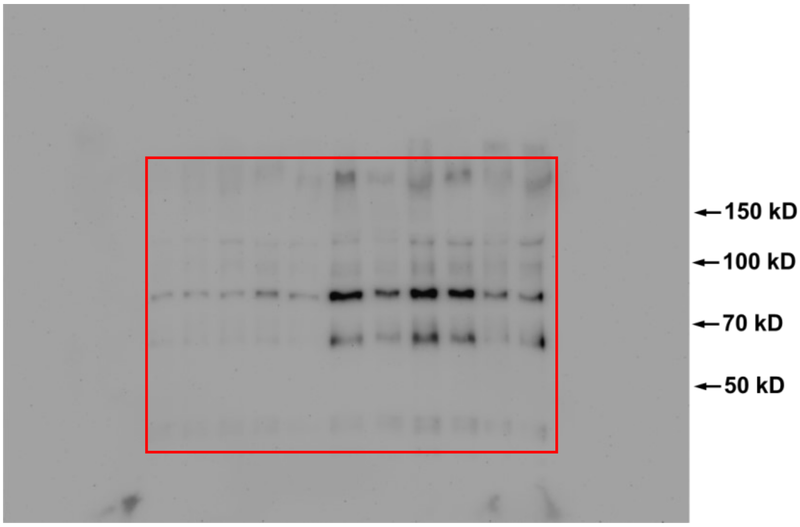

Supplement: Supplementary file 2 — Fig S2 [file PHY2-9-e14965-s002.pdf]
